# Complete Genome Sequence of Dengue Virus Serotype 4 from Guangzhou, China

**DOI:** 10.1128/genomeA.00299-13

**Published:** 2013-05-30

**Authors:** Zhijun Bai, Qi Liu, Li-yun Jiang, Li-cheng Liu, Yi-min Cao, Yang Xu, Qin-long Jing, Lei Luo, Zhi-cong Yang, Yong-qiang Jiang, Wei-jun Chen, Biao Di

**Affiliations:** Guangzhou Center for Disease Control and Prevention, Guangzhou, Guangdong, Chinaa; State Key Laboratory of Pathogen and Biosecurity, Institute of Microbiology and Epidemiology, Academy of Military Medical Sciences, Beijing, Chinab; BGI in Shenzhen, Shenzhen, Chinac

## Abstract

In 2010, the first complete genome sequence of a dengue virus serotype 4 genotype II strain was reported in Guangzhou, China. Here, we report another isolated strain belonging to the genotype II. Our results will offer help to dengue virus control and precautions.

## GENOME ANNOUNCEMENT

Dengue virus (DENV) is a globally prevalent arthropod-borne virus that commonly infects humans. DENV is a positive single-stranded RNA virus and belongs to the genus *Flavivirus* within the *Flaviviridae* family (1). DENV transmission often occurs in urban tropical and subtropical areas, such as Southeast Asia, the Pacific, and the Americas. DENV contains four distinct serotypes (DENV-1 to -4) and can cause dengue fever (DF), dengue hemorrhagic fever (DHF), and dengue shock syndrome (DSS) (2, 3). Approximately 2.5 billion people are at risk of infection, and 50 to 100 million infections occur each year, resulting in >25,000 deaths (4). Dengue virus has four serotypes (DENV-1, DENV-2, DENV-3, and DENV-4). DENV-4 is classified into four genotypes (5). In past decades, DENV-4 appeared in many areas, including Thailand, Singapore, Indonesia, Brazil, and India (6–8). In China, Guangdong Province is one of the major DENV-affected areas. DENV-4 was first identified at Foshan in 1978, and it reoccurred in Guangzhou by an imported case in 2002 (9). A local outbreak of DF was confirmed in Guangzhou, China, in 2010, and the agent was identified as DENV-4 genotype II. In this study, another genotype DENV-4 was described.

A dengue virus 4 (DENV-4) was isolated from a 40-year-old female DF patient on 25 July 2012, 6 days after the onset of symptoms. Total RNA was extracted from viral culture in C6/36 cells using the RNeasy mini kit (Qiagen). Viral cDNA synthesis was produced by SuperScript II reverse transcriptase (Invitrogen) with a reverse primer, and the complete genome sequence was obtained by PCR and sequencing. The genome was assembled using DNASTAR 5.0. The length of this isolated virus is 10,574 bp. Phylogenetic analysis based on the nucleic acid sequence of the complete envelope gene and genome was conducted using the neighbor-joining method in MEGA 5.04 software. The results showed that this strain (GZ/9809/2012) belongs to genotype II and has a close relationship with the Southeast Asian strains, including DENV-4SG/06K2270DK1 from Singapore, THD4_0734_00 from Thailand, and SW38i from Indonesia. Compared with those strains, the amino acid homologies of this strain are 99.6%, 98.7% and 98.6%, respectively. The DENV-4 strain of GZ30 isolated from Guangzhou in 2010 also belongs to genotype II. Compared to this strain, the amino acid homology of the GZ/9809/2012 strain is 98.8%. The strain described here was possibly imported from Southeast Asia, most likely from Singapore.

In past decades, the number of regions of DENV virus infection has increased dramatically. This is probably due to the poorly planned urbanization of many developing countries and the rapid spread of serotypes through global human travel networks (10). The report of GZ/9809/2012 described here will offer help with disease control and precautions.

### Nucleotide sequence accession number.

The complete genome sequence of dengue virus serotype 4 strain GZ/9809/2012 has been submitted to GenBank under the accession no. KC333651.
